# Consistent Condom Use during Casual Sex among Long-Truck Drivers in Togo

**DOI:** 10.1371/journal.pone.0153264

**Published:** 2016-04-12

**Authors:** Issifou Yaya, Dadja Essoya Landoh, Bayaki Saka, Kokou Vignikin, Abdoul-Samadou Aboubakari, Kouamé Mathias N’dri, Kodjo Dodji Gbetoglo, Atavi-Mensah Edorh, Komla Ahlegnan, Holali Comlan Yenkey, Ayawavi Sitsopé Toudeka, Palokinam Pitché

**Affiliations:** 1 Laboratoire de Santé Publique (EA 3279), Faculté de Médecine Campus Timone, Aix-Marseille Université, Marseille, France; 2 Unité de Recherche Démographique (URD) Université de Lomé, Lomé, Togo; 3 Division de l’épidémiologie, Ministère de la santé du Togo, Lomé, Togo; 4 Service de dermatologie et IST, CHU Sylvanus Olympio, Université de Lomé, Lomé, Togo; 5 Service de gynéco-obstétrique, Centre Hospitalier Universitaire de Kara, Kara, Togo; 6 Conseil National de Lutte contre les IST/VIH/Sida, Lomé, Togo; Kasturba Medical College Manipal, Manipal University, INDIA

## Abstract

**Background:**

In 2008, the proportion of truck drivers who were not systematically protected during sex was 63% with casual partners and 60% with sex workers. Despite the high level of knowledge on HIV/AIDS and the growing awareness of the existence of the risk of HIV infection, condom use always encounters resistance among truck drivers in Togo. We sought to document the factors associated with condom use during casual sex among trucks’ drivers in Togo.

**Methods:**

This was an analytical cross-sectional study conducted in 2010 and targeted truckers at truck station on the two main roads of Togo, Lomé-Cinkassé and Kodjoviakopé-Sanvee Condji.

**Results:**

In this study, 1,782 trucks’ drivers and their helpers were interviewed. All were men, and their mean age was 28.8 ± 8.8 years. Trucks’ drivers were doing an average of 3 stops on their journeys and 1,229 (69%) of them had at least two years of experience in the work. Of the 1,782 trucks’ drivers, only 620 (34.8%) had consistently used condoms during casual sex in the last three months. In multivariate analysis, predictors were: education level (primary schooling: OR = 1.54; *p = 0*.*002;* Secondary schooling and higher OR = 1.38; *p = 0*.*036*), good knowledge of ways of HIV transmission (OR = 1.53; *p = 0*.*000*), tested for HIV (OR = 1.67, *p = 0*.*000*), duration in the profession (2–5 years: OR = 1.43, *p = 0*.*008*; more than 5 years: OR = 1.38, *p = 0*.*027*), and HIV risk’s perception (OR = 1.44, p = 0.000).

**Conclusion:**

These results highlight factors associated with consistent condom use during casual sex by truck drivers in Togo. This is a key population group at high risk of HIV transmission toward which the national HIV/AIDS control program should strengthen the HIV prevention strategies.

## Background

HIV transmission occurs mainly sexually during unprotected sex with an infected individual. The prevalence of HIV infection is particularly high in the vicinity of the main roads and in areas with high rates of displaced persons related to natural disasters and conflicts [1]. For example, the prevalence of HIV among truckers is by location estimated at more than ten times that of the general population [2]. It is well known that truckers have an important role in the spread of sexually transmitted infection and HIV in several countries in the world including in Africa [3–4].

Truck drivers are known as group which is sexually active and their work place offers great opportunities for high risk behaviors for HIV infection. Some studies have reported a high prevalence of sexual risk behavior in this group including sexual multiple partners and low rates of consistent condom use [3–8]. In fact, in North India, recent findings have shown that almost 30% of drivers and 50% of helpers didn’t use condom when having sexual contact with commercial sex workers [7], exposing themselves to HIV infection. In 2012, a study conducted on the main roads in Uganda reported that only 21% of truck drivers have consistently used condoms during sexual intercourse during their journeys [5]. In another study conducted in Nigeria, nearly three-quarters (74.3%) of the truck drivers had multiple irregular or occasional sexual partners [6]. This sexual behavior, having multiple sexual partners or casual sex, could facilitate more the HIV spread in the general population.

In Togo, no nationwide HIV prevalence study has been conducted among transport workers; however, a behavioral study conducted in 2008 has shown, that the prevalence of high-risk sexual behaviors was high among this key population [9]. In 2008, the proportion of truckers who have used consistently condom during sex with casual partners was 63% and 60% with sex workers. Despite the high level of knowledge on HIV/AIDS and the growing awareness of the existence of the risk of infection, condom use always knows resistance in truckers in Togo [9]. In order to have reliable, contextualized and updated information on condom use for planning interventions against HIV/AIDS among truckers, it seemed appropriate to document the prevalence and factors associated with consistent condom use in casual sex in this Most at Risk population (MARPs) in Togo.

## Method

### Study population

This study targeted long-truck drivers, aged more than 15 years and operating in Togo. Long-distance truck drivers were defined as truckers traveling to destinations away from their point of origin and who crossed Togo’s borders. The survey was undertaken at eleven transshipment locations along the two main routes of the country Lomé-Cinkassé and Kodjoviakopé-Sanvee Condji.

### Data sources

This was a cross-sectional behavioral and nationwide survey conducted in 2010 among long-distance truckers in Togo on the two main migratory roads, Lomé-Cinkassé and Kodjoviakopé-Sanvee Condji as part of the STI/HIV/AIDS prevention Project on Migratory Roads of West Africa (PSAMAO = Prévention du SIDA sur les Axes Migratoires de l'Afrique de l'Ouest). These two roads were chosen because they are the two main roads of Togo; the first road Lomé-Cinkassé traversing the country from South to North and the second road Kodjoviakopé-Sanvee Condji going from West to East of Togo.

### Data collection

The study was carried out using an established protocol validated by the national reference group for monitoring and evaluation and research of the Togolese National AIDS and sexually transmitted infections control Council (CNLS-IST). In total six supervisors and forty one interviewers were recruited and trained for five days on the behavioral aspect and data collection process.

Data were collected from February 13 to 19 at 11 truck stands or goods load place along the two main roads. In total, 1,782 truckers were interviewed. Behavioral data, including information about demographics, work, sexual partners, and condom use, were collected through face-to-face interviews. Each individual has been questioned about his condoms use during casual sex in the last three months as well as the status of their HIV testing before the survey.

Sex was considered as casual in this study if the trucker had sex with partners other than his wife for married truckers or other than the regular partner (stable sexual partner) for singles, divorced or widowed truckers. It was coded “1” if the trucker had consistently used condom and “0” otherwise.

Consistent condom use was defined as “using condoms during each sexual intercourse”. Condom use was considered as consistent when trucker reported using condoms at each sexual intercourse during the last three months before the survey.

The number of truck stops was considered as the number of times the trucker stopped during his round trip HIV risk perception or self-perception of HIV risk is defined as the level of awareness of the participant towards HIV infection risk and was assessed using the following question: “according to your sexual behavior and your life’s style, are you at risk to be infected by the HIV?

### Ethical consideration

The entire study protocol was approved by the board committee of the Bioethics Committee of the Ministry of Health and the National council for HIV/AIDS Control. The participants gave their written informed consent after the verbal explanation of the aim of the study. For participants under 18 years old (minors) who were enrolled in this study, a written informed consent form was signed by their parents or guardians, the truckers. For each of the participant included in the survey, the objectives, benefits to participate in the survey and progress of the investigation were clearly stated as well as their right to interrupt the interview without justification. Participants were given information on safe sex practices and HIV prevention as well as information on care facilities available in the area. Data were recorded in an anonymously linked manner using numerically coded cards.

### Data analysis

The collected data were entered by trained officers using EPI-DATA 3.1 software. Analysis was performed using SPSS Inc. version 17.0 software (SPSS Inc., Chicago, IL, USA). In the univariate analysis, for continuous variables, means and standard deviation were calculated while for categorical variables we calculated proportions. Our main outcome variable was consistent condoms use during the last three months compare to non consistent condoms use. The chi-square test or Fisher´s exact test were used when appropriate in bivariate analysis. Multivariate backwards stepwise logistic regression analysis was performed to identify independent risk factors for the dichotomous outcome consistent condoms use and non consistent condoms use. The variables significant during bivariate analysis at a p-value less than 0.05 and uncorrelated were then selected in an initial model of logistic regression to assess their contribution to the consistent use of condoms among truckers by estimating the adjusted odds ratios (ORs) and their 95% confidence intervals (CI).

## Results

At the 11 truck stands targeted, 1782 truckers were interviewed. The mean age of the respondents was 28.8±8.8 years, ranging from 15 to 73 years. Of the 1,782 truckersinterviewed1, 357 (76.1%) were less than 35 years old. They perform on average 3 stops on a taken road during their journey. One thousand and twenty-five (57.5%) of the 1782 truck drivers were not in couple, 855 (48%) of them had at least secondary school level and 1,229 (69%) of them had at least two years of experience in the work. In total, for 1,283 (72%) truckers it has taken them less than two weeks for a round trip. Of the 1,782 truck drivers, 860 (48.3%) were aware that they were at risk of contracting HIV while 845 (47.4%) had already done their HIV testing (Table 1).

**Table 1:** Sociodemographic and work related characteristics of truck drivers in Togo, 2010.

Characteristics	Number of truckers N (%)	Consistent condom use n (%)	OR	p-value
**Age**	* *	* *		**<0.001**
**Less than 25 years**	*660 (37*.*0)*	*211 (32*.*0)*	1	
**25–34 years**	*697 (39*.*1)*	*287 (41*.*2)*	1.49 [1.19 ; 186]	
**More than 35 years**	*425 (23*.*9)*	*122 (28*.*7)*	0.86 [0.66 ; 1.12]	
**Marital status**				**0.025**
**Single / Divorced / Widowed**	*1025 (57*.*5)*	*353 (34*.*4)*	1	
**Married monogamous**	*594 (33*.*3)*	*224 (37*.*7)*	1.15 [0.93 ; 1.42]	
**Married polygamous**	*163 (9*.*2)*	*43 (26*.*4)*	0.68 [0.47 ; 0.99]	
**Education**				**0.002**
**None**	*374 (21*.*0)*	*103 (27*.*5)*	1	
**Primary**	*553 (31*.*0)*	*213 (38*.*5)*	1.65 [1.24 ; 2.19]	
**Secondary and higher**	*855 (48*.*0)*	*304 (35*.*6)*	1.45 [1.11 ; 1.89]	
**Standard of living**				0.355
**Low**	*651 (36*.*5)*	*228 (35*.*0)*	1	
**Medium**	*733 (41*.*1)*	*265 (36*.*2)*	1.05 [0.84 ; 1.31]	
**High**	*398 (22*.*3)*	*127 (31*.*9)*	0.87 [0.67 ; 1.13]	
**Level of knowledge**				**<0.001**
**Partial**	*646 (36*.*3)*	*186 (28*.*8)*	1	
**Complete**	*1136 (63*.*7)*	*434 (38*.*2)*	1.53 [1.24 ; 1.88]	
**Level of exposure to information sources**				0.076
**Less exposed**	*1033 (58*.*0)*	*377 (36*.*5)*	1	
**More exposed**	*749 (42*.*0)*	*243 (32*.*4)*	0.84 [0.69 ; 1.02]	
**Tested for HIV**				**<0.001**
**Yes**	*845 (47*.*4)*	*351 (41*.*5)*	1.76 [1.45 ; 2.14]	
**No**	*937 (52*.*6)*	*269 (28*.*7)*	1	
**Duration in the driver profession**				**0.002**
**Less than 2 years**	*553 (31*.*0)*	*161 (29*.*1)*	1	
**2 to 5 years**	*519 (29*.*1)*	*203 (39*.*1)*	1.56 [1.21 ; 2.01]	
**Over 5 years**	*710 (39*.*9)*	*256 (36*.*1)*	1.37 [1.08 ; 1.74]	
**Frequency of the road trips**				0.537
**Every 2 weeks**	*1223 (68*.*6)*	*420 (34*.*3)*	1	
**Each month**	*501 (28*.*1)*	*176 (35*.*1)*	1.04 [0.84 ; 1.29]	
**Every 3 months**	*58 (3*.*3)*	*24 (41*.*4)*	1.35 [0.79 ; 2.31]	
**Duration of round trip**				0.132
**Less than 2 weeks**	*1283 (72*.*0)*	*460 (35*.*9)*	1	
**More than 2 weeks**	*499 (28*.*0)*	*160 (32*.*1)*	0.84 [0.67 ; 1.05]	
**Length of stay at destination**				**0.048**
**Within a week**	*1579 (88*.*6)*	*562 (35*.*6)*	1	
**Over one week**	*203 (11*.*4)*	*58 (28*.*6)*	0.72 [0.52 ; 0.99]	
**Number of stops during the trip**				0.105
**0 or 1**	*116 (6*.*5)*	*42 (36*.*2)*	1	
**2 or 3**	*1261 (70*.*8)*	*420 (33*.*3)*	0.88 [0.59 ; 1.31]	
**4 or more**	*405 (22*.*7)*	*158 (39*.*0)*	1.13 [0.74 ; 1.73]	
**Risk perception of HIV infection**				**<0.001**
**Yes**	*860 (48*.*3)*	*342 (39*.*8)*	1.53 [1.26 ; 1.86]	
**No**	*922 (51*.*7)*	*278 (30*.*2)*	1	
**Consistent condom use**				
**Yes**	*620 (34*.*8)*	*-*	*-*	*-*
**No**	*1162 (65*.*2)*	*-*	*-*	

Of the 1,782 truckers interviewed, 1,247 (70%) had multiple sexual partners, and 620 (34.8%) of them had consistently used condoms during casual sex during the last three months (Table 1).

In the bivariate analysis, the consistent condom use was associated with the age of the trucker (p <0.001), the marital status (p = 0.025), the level of education (p = 0.002), the knowledge of the ways of HIV transmission (p <0.001), the HIV testing (p <0.001), the duration in the profession (p = 0.002), duration of stay at destination (p = 0.048) and the HIV risk’s perception (p <0.001) (Table 1).

In multivariate analysis, five (05) factors remained associated with consistent condom use during casual sex among truckers. In fact educated truck drivers (primary OR = 1.56, p = 0.002; secondary and over OR = 1.34, p = 0.036), those with a good knowledge of ways of HIV transmission (OR = 1.53, p = 0.000), those who were tested for HIV (OR = 1.67, p = 0.000), those with at least two years of duration in the profession (2 to 5 years: OR = 1.43, p = 0.008; more than 5 years: OR = 1.38, p = 0.027) and those with a good perception of risk of HIV infection (OR = 1.44, p = 0.000) were more likely to use condoms consistently during casual sex (Table 2).

**Table 2:** Multiple logistic regression of factors associated with consistent use of condom among truck drivers in Togo, 2010 (N = 1782).

Characteristics	aOR	95% CI for aOR	p-value
**Age**			
**Less than 25 years**	**Ref**	-	-
**25–34 years**	**1.25**	[0.96 ; 1.62]	0.094
**More than 35 years**	**0.78**	[0.54 ; 1.14]	0.198
**Marital status**			
**Single / Divorced / Widowed**	**Ref**	-	-
**Married monogamous**	**1.1**	[0.85 ; 1.44]	0.468
**Married polygamous**	**0.77**	[0.49 ; 1.22]	0.266
**Education level**			
**None**	**Ref**	-	-
**Primary school**	**1.56**	[1.18 ; 2.11]	**0.002**
**Secondary and higher**	**1.34**	[1.02 ; 1.77]	**0.036**
**Level of knowledge of HIV/AIDS**			
**Partial**	**Ref**	-	**-**
**Complete**	**1.53**	[1.23 ; 1.89]	**<0.001**
**Tested for HIV**			
**No**	**Ref**	-	**-**
**Yes**	**1.67**	[1.36 ; 2.05]	**<0.001**
**Duration in the driver profession**			
**Less than 2 years**	**Ref**	-	-
**2 to 5 years**	**1.43**	[1.10 ; 1.87]	**0.008**
**Over 5 years**	**1.38**	[1.04 ; 1.84]	**0.027**
**Length of stay at destination**			
**Within a week**	**Ref**	-	-
**Over one week**	**0.74**	[0.53 ; 1.04]	0.081
**HIV risk perception**			
**No**	**Ref**	-	**-**
**Yes**	**1.44**	[1.17 ; 1.76]	**<0.001**

Pseudo-R^2^ of the regression model = 10.3%

## Discussion

The results of this study identified five factors that were associated with consistent use of condoms among the truckers when having casual sex in the last three months. Our results are similar to those of other studies conducted in some developing countries on similar population groups [2–4, 6–8, 10–12].

In our study, only 34.8% of surveyed drivers had consistently used a condom during casual sex in the last three months. Although this rate always low, a tendency to increase of the consistent use of condom in this group targeted by PSAMAO’s Project is noted since 2008. In 2008, a behavioral study conducted in a similar group reported that the rate of consistent condom use was 30.1%. The rate in the current study is close to that of 21% reported in Uganda on two main roads of the country [5], and 19.1% reported in India [11]. This low rate of condom use among truck drivers could partly explains the very high prevalence of HIV infection in this population group compared to the general population [2]. Contrary to these studies, a study conducted at a parking site at Kenya and Uganda border reported a satisfactory rate of 70% of condom use among truck drivers [13].

Concerning the identified factors, it appears that, first, the truck drivers with a high level of education have consistently used condom during casual sex in the last three months. Our results are similar to those found by a Tanzanian study where truck drivers with a high level of education had a strong propensity to use condoms during casual sex [10]. The explanation we propose is that education promotes an understanding of prevention messages driven by the education and information on intervention strategies. Also, the high level of knowledge of the routes of transmission of HIV infection has a positive influence on consistent condom use among truck drivers. Our results confirm those reported in Bangladesh [3] and in India [11] which stated that the truck driver, who had heard of HIV/AIDS, with a high level of knowledge about preventive practices of HIV infection were more likely to use condoms with sexual partners.

It appears that the level of exposure to sources of information remains a very important factor for acquiring new knowledge related to socio-cultural, economic, and political or health phenomena or problems, leading to better prevention strategies development.

Furthermore, our study found that truck drivers who were tested for HIV, and are aware of their HIV status were about 1.7 times more likely to use condoms consistently. Conducting HIV testing with pre and post-test counseling is considered as effective means of preventing HIV infection. Other authors in Togo and in other developing countries have demonstrated the effectiveness of the voluntary counseling and anonymous test in reducing sexual risk behaviors [14–16]. It is therefore important to create at the trucks stands on the roads, counseling centers and HIV testing for truck drivers.

Likewise in our study, other authors have noted that the truck drivers who had a good perception of risk of HIV infection were more likely to protect themselves by using consistently condom during casual sex compare to those who had poor perception of HIV infection risk [10, 12]. The perception of risk of HIV infection leads individuals to adopt safe sexual behavior. Lastly, for the duration in the profession our results are similar to those of Gibney et *al*. in Bangladesh [3]; and Dude et *al*. in India [11] who reported that the number of years of working in transport sector, is a protective factor against the exposure risk to STIs, including HIV.

## Conclusion

This study documents factors associated with consistent condom use during casual sex among truck drivers in Togo. Truckers remain a risky group in which the prevalence of consistent condom use during casual sex is low. The level of education, good knowledge of ways of HIV transmission, testing for HIV, duration in the profession, and HIV risk’s perception are the main predictors of consistent condom use during casual sex. These data demonstrated that there are needs for HIV/AIDS control program to strengthen HIV prevention interventions among this Most at Risk population of HIV transmission.
